# To kill a microRNA: emerging concepts in target-directed microRNA degradation

**DOI:** 10.1093/nar/gkae003

**Published:** 2024-01-15

**Authors:** Amber F Buhagiar, Benjamin Kleaveland

**Affiliations:** Department of Pathology and Lab Medicine, Weill Cornell Medicine, New York, NY10065, USA; Department of Pathology and Lab Medicine, Weill Cornell Medicine, New York, NY10065, USA

## Abstract

MicroRNAs (miRNAs) guide Argonaute (AGO) proteins to bind mRNA targets. Although most targets are destabilized by miRNA–AGO binding, some targets induce degradation of the miRNA instead. These special targets are also referred to as trigger RNAs. All triggers identified thus far have binding sites with greater complementarity to the miRNA than typical target sites. Target-directed miRNA degradation (TDMD) occurs when trigger RNAs bind the miRNA–AGO complex and recruit the ZSWIM8 E3 ubiquitin ligase, leading to AGO ubiquitination and proteolysis and subsequent miRNA destruction. More than 100 different miRNAs are regulated by ZSWIM8 in bilaterian animals, and hundreds of trigger RNAs have been predicted computationally. Disruption of individual trigger RNAs or *ZSWIM8* has uncovered important developmental and physiologic roles for TDMD across a variety of model organisms and cell types. In this review, we highlight recent progress in understanding the mechanistic basis and functions of TDMD, describe common features of trigger RNAs, outline best practices for validating trigger RNAs, and discuss outstanding questions in the field.

## Graphical Abstract

**Graphical Abstract:**
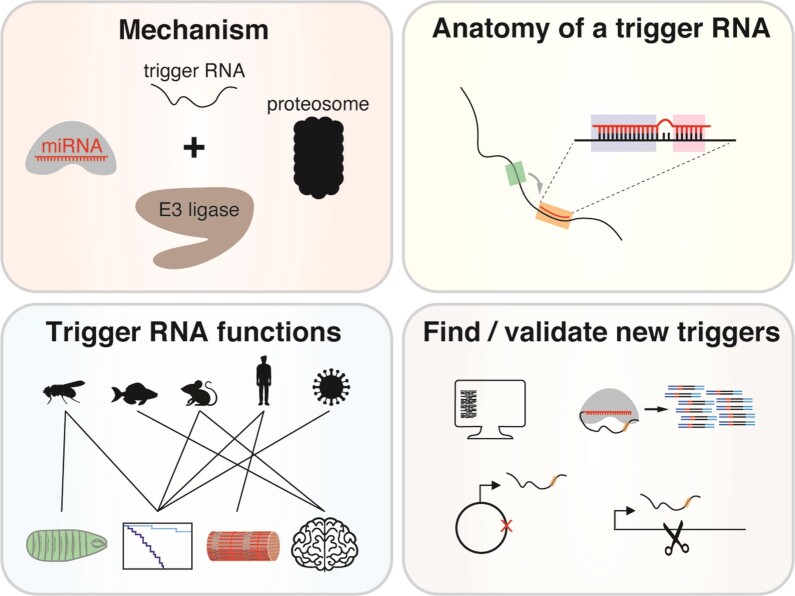

## Introduction

**Figure 1.**
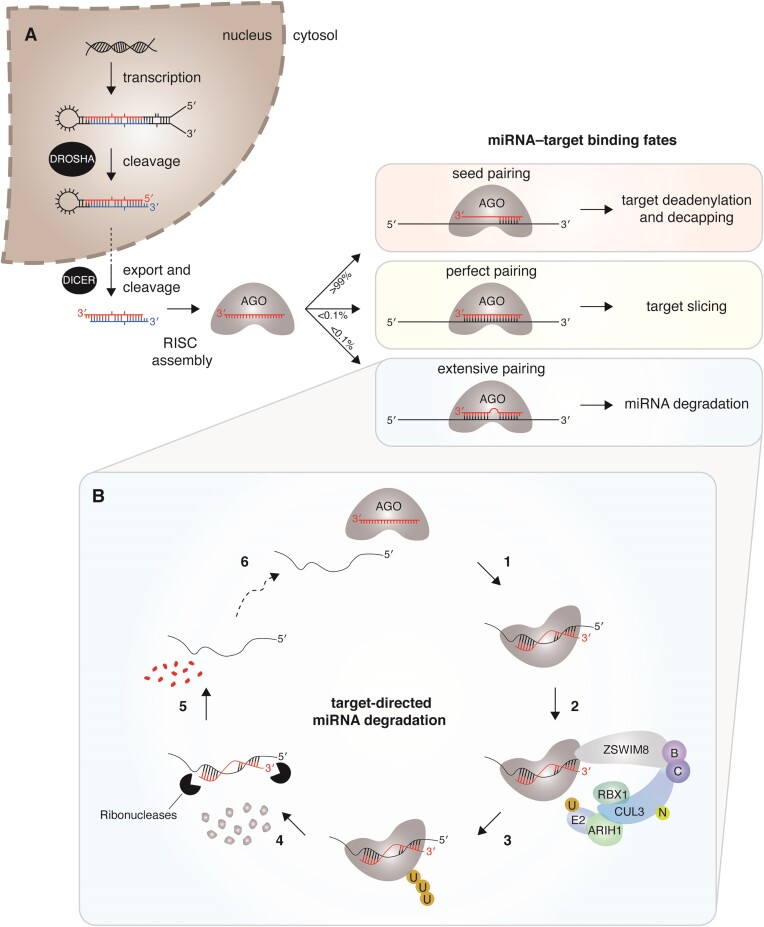
The miRNA life cycle. **(A)** miRNA biogenesis and targeting are depicted. After sequential processing in the nucleus and cytosol by DROSHA/Microprocessor and DICER, respectively, the miRNA duplex is loaded into the AGO effector protein and one strand is removed. The fate of the AGO–miRNA complex and target RNA depend on the extent of base-pairing between miRNA and target. These outcomes include target degradation via deadenylation and decapping, target degradation via endonucleolytic cleavage (also known as slicing), and miRNA degradation. **(B)** In the current model of target-directed miRNA degradation, extensive complementarity between the AGO–miRNA complex and target RNA induces a stabilized ternary complex (1), which recruits the ZSWIM8 substrate adapter, other subunits of the E3 ubiquitin ligase including neddylated (N) CUL3, ELONGINS B and C, ARIH1, and RBX1, and an ubiquitin-conjugating enzyme (E2) (2), leading to polyubiquitination (U) (3) and proteasomal degradation of AGO (4), followed by degradation of the deprotected miRNA (5). No longer bound to miRNA, the target RNA can interact with a new AGO–miRNA complex (6), initiating another round of miRNA degradation.

MicroRNAs (miRNAs) remodel the transcriptome by accelerating the degradation of specific messenger RNAs. miRNAs are produced from larger transcripts that undergo sequential, tightly regulated processing to generate a 20–24 nucleotide (nt) miRNA duplex (Figure 1A). The duplex is then loaded into an Argonaute (AGO) protein and the passenger strand is removed, after which the mature miRNA guides AGO to sites in the 3′ untranslated regions (UTRs) of target RNAs. The binding of the AGO–miRNA complex and its association with the GW182/TNRC6 family of scaffolding proteins leads to increased recruitment of deadenylases and decapping machinery to target RNAs. With hundreds of conserved miRNAs, each with tens to hundreds of conserved mRNA targets (1), the influence of miRNA-directed mRNA degradation is pervasive in both development and disease. The biogenesis, targeting, and biological functions of miRNAs have been the subject of recent reviews (2–4) and therefore will not be discussed further. Instead, we focus on miRNA degradation, which has emerged as an important mechanism by which miRNA levels are differentially regulated and marks an exciting new chapter in our understanding of miRNAs.

With half-lives that typically exceed 12–24 h, miRNAs are many times more stable than mRNAs (5–11). This stability is attributed to AGO, which protects both ends of the miRNA and much of its phosphate backbone from nucleolytic attack (12–19). Nevertheless, in any transcriptome-wide miRNA half-life study, a small fraction (∼5%) of miRNAs have shorter half-lives (<12 h) (5–11). In addition, when cells are treated with growth factors, cytokines, serum or other stimuli, some miRNAs disappear rapidly (20–24). Whether this decrease arises due to induced degradation or reduced production combined with constitutive degradation, the fact remains that degradation is required for shaping the levels and dynamics of miRNAs in diverse biological settings.

Over the years, a variety of nucleases have been implicated in miRNA degradation, including 5′→3′ exonucleases XRN1 and XRN2, 3′→5′ exonucleases SDN, PARN, PNPT1 and the exosome complex, and the TSN endonuclease (5,25–32). However, all these nucleases have activity towards more than one type of RNA, which makes it difficult to demonstrate that any increase in miRNA abundance after *in vivo* depletion of the nuclease is a direct effect. Furthermore, most of these nucleases have little to no sequence preference, a characteristic that is hard to reconcile with findings that only a subset of miRNAs have short half-lives, unless these nucleases cooperate with other proteins that provide specificity (30). But the biggest conundrum is how these nucleases might access their substrates if the miRNAs are still loaded in AGO. While all the nucleases mentioned above show *in vitro* activity towards single-stranded RNA, only two, XRN2 and TSN, have *in vitro* activity towards single-stranded RNA loaded in AGO protein. XRN2 appears to promote unloading of the miRNA and subsequent degradation (25), whereas the TSN endonuclease preferentially cleaves the miRNA backbone at CA and UA dinucleotides that are likely exposed to solvent (32). Of note, both XRN2 and TSN activity are inhibited by target binding to AGO–miRNA complexes (25,32), underscoring the important role that target RNAs play in limiting some types of miRNA degradation.

On the other hand, some targets induce degradation of specific miRNAs, a process called target-directed miRNA degradation (TDMD). Whereas miRNA-directed mRNA degradation is achieved through Watson–Crick base-pairing of the seed region, nucleotides 2–8 of the miRNA (counting from the 5′ end), all known examples of TDMD rely both on seed pairing and extensive pairing 3′ of the seed region between the miRNA and its target. These unusual targets are also referred to as trigger RNAs because they trigger degradation of specific miRNAs.

In this review, we provide a brief history of TDMD, describe recent progress on understanding the mechanism underlying TDMD, discuss common features of trigger RNAs, and propose a set of standards and assays important for functional validation of candidate miRNA–trigger RNA pairs. We conclude by highlighting unanswered questions and key challenges in the field.

## The ZSWIM8 E3 ubiquitin ligase mediates TDMD

### An unexpected mechanism of miRNA degradation

TDMD was first reported in two landmark studies which demonstrated that expression of viral and artificial RNAs in mammalian and Drosophila cell lines causes the destruction of specific miRNAs (33,34). The common feature uniting these TDMD-inducing RNAs is a highly complementary miRNA binding site. Later, additional viral and non-viral transcripts were also shown to induce degradation of specific miRNAs (35–40). One of these transcripts, an endogenous long noncoding RNA (lncRNA) called *Cyrano*, drives the degradation of its substrate miR-7 with remarkable potency, eliminating more than 98% of the miRNA in some mouse tissues (40).

As AGO-mediated protection of the 5′ and 3′ ends of the miRNA is thought to drive miRNA stability, the prevailing model for specific miRNA degradation involved target-mediated deprotection of the 3′ end. This deprotection would lead to the addition of untemplated nucleotides (tailing) and removal of nucleotides (trimming), followed by eventual degradation of the entire miRNA. This model was supported by evidence of increased tailing and/or trimming of miRNAs in the presence of highly complementary targets (33,36,38–42), structures of the AGO–miRNA complex bound to highly complementary targets that show release of the miRNA 3′ end from the PAZ domain of AGO, and findings that tailing of pre-let-7 and other noncoding RNAs (ncRNAs) acts as a signal for 3′-to-5′ exonucleolytic decay (43–45). Nonetheless, the protein factors required for TDMD remained elusive and depletion of specific trimming and tailing factors did not stabilize miRNAs regulated by TDMD (40,46).

A breakthrough came in 2020: using genome-wide CRISPR screens for factors that facilitate *CYRANO*-directed miR-7 degradation, two groups identified Zinc Finger SWIM-Type Containing 8 (ZSWIM8), the substrate-binding subunit of a Cullin-RING E3 ubiquitin ligase, and showed that ZSWIM8 and the ubiquitin-proteasome pathway are essential mediators of TDMD (46,47). These results led to a new model, whereby highly complementary targets (trigger RNAs) induce a conformational change in the AGO–miRNA complex that is recognized by ZSWIM8, resulting in ubiquitination and subsequent proteasomal degradation of AGO (Figure 1B). No longer protected by AGO, the miRNA is susceptible to one or more exonucleases. Once the miRNA is degraded, the trigger RNA can bind and trigger destruction of another AGO–miRNA complex, such that the trigger acts with multiple-turnover. In addition, both groups showed that tailing and trimming are dispensable; miR-7 with a 3′-terminal 2′-*O*-methyl group is resistant to tailing and trimming, but still susceptible to TDMD (46,47). Although many features of this model have experimental support, some questions remain: What is the degron that ZSWIM8 recognizes? How is the miRNA released and which nucleases are responsible for miRNA degradation? What role could deubiquitinases have in limiting ZSWIM8 activity and TDMD?

### ZSWIM8: conservation, structure and substrates

**Figure 2.**
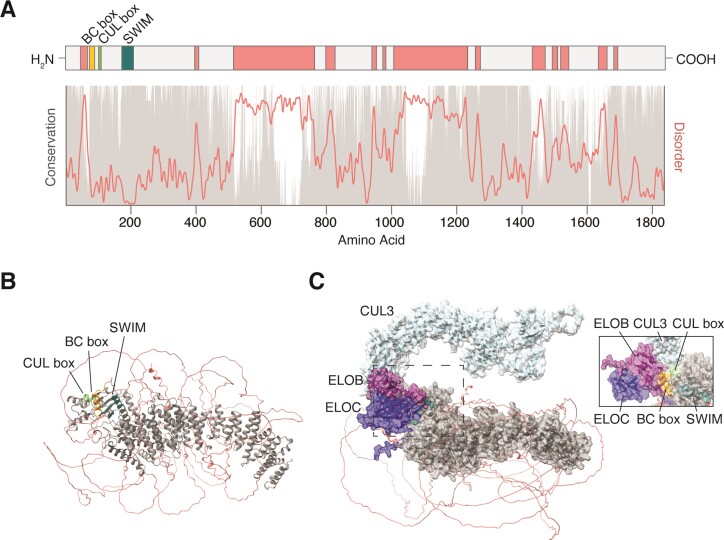
ZSWIM8 protein structure and conservation. (A) Linear diagram of the ZSWIM8 protein showing the size and location of the BC box (orange), CUL box (green), and SWIM domain (teal), as well as several regions of predicted disorder (coral). Below, conservation scores (gray) derived from CONSURF-DB (105) and disorder scores (coral) derived from IUPRED (104) have been plotted along the length of ZSWIM8. In general, intrinsically disordered regions are poorly conserved. (B) The AlphaFold prediction (AF-A7E2V4-F1) of human ZSWIM8 protein is shown (107,108). The BC box (orange), CUL box (green), and SWIM domain (teal) are tightly clustered at one end of the protein and accessible to solvent and other proteins. The conserved protein core (gray) consists of repeating alpha helices that form a solenoid-like structure. The disordered regions (coral) are not confidently predicted. (C) AlphaFold-Multimer prediction (via COSMIC) for ZSWIM8 in complex with ELOB (purple), ELOC (navy), and CUL3 (light blue)(109). The inset image provides another look at the ELOB–CUL3–ZSWIM8 interface from a different angle.

In humans, *ZSWIM8* is comprised of 26 exons which are transcribed, processed and translated into an ∼1837 amino acid (AA) protein with three recognizable domains—a BC box (AA 73–88), a Cullin box (AA 100–108), and a SWIM (SWI2/SNF2 and MuDR) domain (AA 174–208)—all clustered at the extreme N-terminus (Figure 2A). *ZSWIM8* orthologs are found throughout Bilateria and possess multiple regions of high conservation across the length of the protein (Figure 2A). Unsurprisingly, *ZSWIM8* is highly constrained against missense mutations and is predicted to be dosage sensitive (48,49), although no disease associations have been reported.

As the substrate receptor of an E3 ubiquitin ligase complex, ZSWIM8 interacts simultaneously with the substrate and with other components of the ligase complex. The BC box binds Elongins B and C (50,51) and the Cullin box is predicted to bind CUL2 (50). However, in human K562 cells, CUL3—not CUL2—co-immunoprecipitates with ZSWIM8 and is required for TDMD (46,47). The Cullin protein scaffolds the substrate receptor, Elongin proteins, and RING-box protein RBX1 into a stable complex, which recruits an E2-conjugating enzyme to the substrate. Interestingly, the role of the SWIM domain, for which ZSWIM8 gets its name, is still poorly understood. In *C. elegans*, the SWIM domain facilitates an interaction with the HSP90 ortholog DAF-21 (51), however, the importance of this interaction in TDMD has not been explored. The remainder of the protein consists of patches of highly conserved and ordered amino acid sequence interspersed by less well conserved and disordered sequence (Figure 2A). Intriguingly, AlphaFold predicts a ZSWIM8 structure consisting of a solenoid-like core of repeated alpha helices surrounded by disordered flexible linkers (Figure 2B) and AlphaFold-Multimer predicts interactions between the ZSWIM8 BC box and Elongins B/C and between the ZSWIM8 Cullin box and CUL3 (Figure 2C). The disordered regions raise the possibility that ZSWIM8 interactions and/or activity may be regulated by phase separation and/or partitioning into biomolecular condensates such as stress granules. Consistent with this idea, exogenous ZSWIM8 is diffusely cytoplasmic under normal growth conditions (52–54), but colocalizes with the stress granule marker G3BP1 in cells treated with inhibitors of either the proteasome or HSP90 (54).

*ZSWIM8* has three paralogs, *ZSWIM4/5/6*, which encode proteins that have a BC box, a Cullin box, and a SWIM domain. However, the functions and substrates of ZSWIM4/5/6 are poorly understood and there is no evidence that these paralogous proteins act redundantly with ZSWIM8. In fact, human *ZSWIM8* is more similar to distant orthologs, *C. elegans ebax-1* (24% amino acid sequence identity) and *D. melanogaster dora* (35% identity), than to any of these paralogs (∼18% identity).

For ZSWIM8 and other ubiquitin ligases, discovering and validating substrates is a major challenge. Interactions between a substrate and its corresponding ligase are usually transient, ubiquitinated species are rapidly degraded, and only a fraction of the total substrate pool may be regulated at a given time. Indeed, the vast majority of AGO is loaded with miRNAs that are not being regulated by TDMD, thus limiting detection of ZSWIM8-dependent changes in endogenous AGO levels, stability, and/or ubiquitination. Fortunately, the levels of endogenous miRNAs can serve as a surrogate for assessing turnover of AGO–miRNA complexes. In addition, Shi et al. and Han et al. used heterologous expression of AGO2 and/or ZSWIM8 to capture interactions between AGO2 and ZSWIM8, identify surface-exposed lysines on AGO2 that are important for TDMD, and demonstrate that TDMD occurs with all four human AGO proteins (46,47).

In addition to AGO proteins, ZSWIM8 also regulates the axon guidance receptor ROBO3/SAX-3 and neuronal signaling protein Dab1 (51,54). It is not yet clear how ZSWIM8 distinguishes each of these substrates and direct evidence is still needed to demonstrate that the regulation currently observed with the heterologous expression of substrates is also observed with the endogenous proteins. As well, ZSWIM8 may have other substrates or E3 ligase-independent functions (52). Thus, any phenotypes ascribed to loss of ZSWIM8 could be due to dysregulation of AGO, accumulation of other substrates, loss of E3 ligase-independent interactions, or some combination thereof.

### Functions of ZSWIM8

Given its extensive conservation, it is not surprising that ZSWIM8 plays important developmental roles in *C. elegans*, *D. melanogaster* and mice. Thus far, 107 miRNAs have been found to be regulated by ZSWIM8 throughout bilateria, including 10 miRNAs in *C. elegans*, 21 in *D. melanogaster*, 73 in mouse embryonic tissues and cell lines and 12 in human cell lines (46,47,55–58). Of the 12 miRNAs identified in human cells, at least 8 are regulated by ZSWIM8 in mice and one of these miRNAs, miR-7, is also regulated in Drosophila.

Worms deficient in ZSWIM8/EBAX-1 are viable but exhibit sluggish locomotion, reduced male mating behavior, impaired egg-laying, and defects in axon guidance (51). Although ZSWIM8 also regulates the turnover of at least 10 miRNAs, including the embryonic miR-35 family, the extent to which miRNA dysregulation contributes to these phenotypes is not yet known (46,59). However, at least a portion of the axon guidance phenotype can be attributed to the role that the ZSWIM8 E3 ligase plays in regulating SAX-3, a ROBO receptor ortholog involved in axon guidance (51). This regulation may be conserved as mouse ZSWIM8 recognizes and targets misfolded human ROBO3 receptor for degradation when both proteins are overexpressed in human cells (51).

In *D. melanogaster*, ZSWIM8/Dora/Pelado deficiency causes embryonic lethality with most embryos failing to hatch into L1 larvae (52,55). This early lethality, which is accompanied by increased expression of 11 miRNAs primarily from the miR-3 and miR-310 family, is partially rescued by reducing levels of miR-3 (55). This result indicates that TDMD is required for proper Drosophila development. Using a mosaic approach to circumvent the early lethality of ZSWIM8 mutants, Molina-Pelayo et al. identified an additional role for ZSWIM8 in regulating the actin cytoskeleton (52). ZSWIM8-deficient *Drosophila* cells have fewer actin-based hairs and filipodia and ZSWIM8-deficient human cells migrate more slowly (52). The authors proposed an E3 ligase-independent role for ZSWIM8 in these phenotypes, but more work is needed to support this conclusion and exclude other contributing factors such as dysregulated miRNAs.

In mice, ZSWIM8 deficiency causes perinatal lethality, likely due to defects in lung sacculation (56,57). Late gestation embryos also have reduced body size and cardiovascular abnormalities, including partially penetrant ventricular septal defects (56,57). More than 50 miRNAs are increased in ZSWIM8-deficient tissues, including 27 in the lung (56,57). miRNA dysregulation contributes to at least one of these phenotypes: the reduced body size of ZSWIM8-deficient embryos is rescued by eliminating the expression of miR-322 and miR-503 (56), members of the miR-15/16 family that are involved in cell cycle regulation (23) and are consistently upregulated across many ZSWIM8-deficient tissues (56,57). The impact that miRNA dysregulation has on lung development and other phenotypes is still under investigation. Whereas conditional deletion of *Zswim8* in cardiac progenitors does not cause lethality (at least up to weaning age) (56), conditional deletion of *Zswim8* in neural and glial progenitors causes partial perinatal and postnatal lethality and the surviving animals have abnormal patterning of the hippocampus and behavioral deficits (e.g. hyperactivity, poor discrimination of novel objects, lack of contextual fear memory) (54). Based on phenotypic similarities between Dab1 mutant mice and conditional ZSWIM8-deficient mice, Wang *et al.* proposed that ZSWIM8 regulates the Reelin-Dab1 pathway during neurogenesis and demonstrated via heterologous expression that ZSWIM8 interacts with and prevents aggregation of Dab1 (54).

### Structural insights from Argonaute–miRNA–trigger complexes

**Figure 3.**
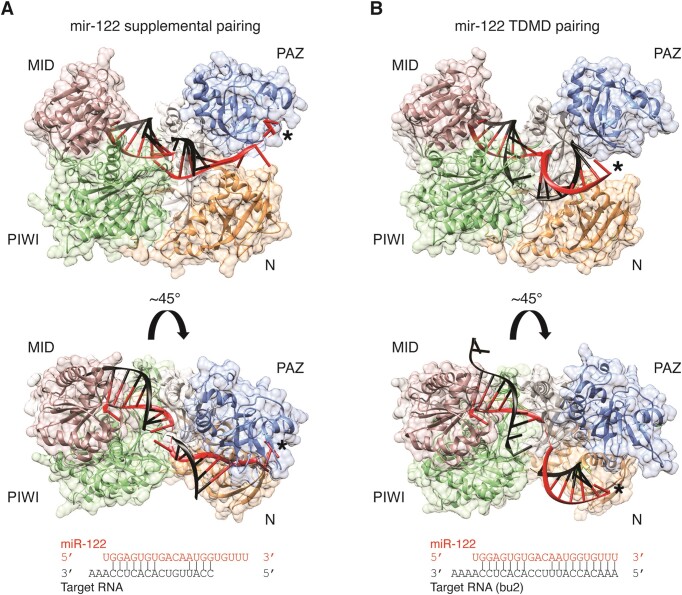
Structures of AGO–miRNA–target and AGO–miRNA–trigger complexes. (A) The structure of human AGO2 (PDB 6N4O, cartoon plus surface representation generated with UCSF Chimera (106)) loaded with miR-122 and bound to a target RNA (black) via seed and 4 nts of supplemental pairing (60). The 5′ end of the miRNA is protected by the MID domain (pink) and the 3′ end of the miRNA (asterisk) is protected by the PAZ domain (blue). The central region of the miRNA is unpaired and the corresponding target nts are not visible in this structure. (B) The structure of human AGO2 (PDB 6NIT, cartoon plus surface representation generated with UCSF Chimera) loaded with miR-122 and bound to a TDMD site (bu2) with 10 nts of 3′ pairing (black) (15). The 5′ end of the miRNA is still protected by the MID domain (pink), however, the 3′ end of the miRNA (asterisk) is no longer protected by the PAZ domain (blue). The 3′ half of the miRNA has rotated, and the sugar-phosphate backbone is no longer intimately associated with AGO. A similar structure was solved with AGO2, miR-27a, and the HSUR1 TDMD site (15).

The mechanisms by which ZSWIM8 distinguishes low-abundance AGO–miRNA–trigger complexes from the multitude of AGO–miRNA–target complexes in cells are not well understood. However, there is evidence that these two types of complexes are structurally distinct. Crystal structures have been solved for human AGO2–miRNA bound to targets with different amounts of base-pairing—seed pairing (12), seed pairing with 4 nt of supplemental 3′ pairing (60), and seed pairing with 9–10 nt of 3′ pairing, as seen with some TDMD triggers (15). Structures of AGO2–miRNA bound to a seed only target or a seed plus supplemental target are very similar, however, clear differences emerge when compared to structures of AGO2–miRNA bound to a trigger RNA (Figure 3A, B). As anticipated, these structures demonstrate that the miRNA binds more extensively to a trigger RNA, which extracts the 3′ end of the miRNA from its binding pocket in the PAZ domain. This extensive pairing is facilitated by a kink in the miRNA–trigger RNA duplex that arises due to unpaired nucleotides between the seed and 3′ pairing. These structures also reveal a conformational change in AGO2 that accompanies release of the 3′ end of the miRNA—the PAZ and N domains rotate ∼8 Å, and the central cleft separating the seed and supplemental pairing chamber widens, although not enough to enable central pairing to the miRNA. Modeling of a perfect duplex into this structure predicts steric clashes between AGO2 and the RNA duplex after the 11th base pair (15), which is consistent with a recent study showing that additional conformational changes are required for full pairing between a miRNA and target (17).

Crystal structures provide static views of distinct conformations but do not reveal the dynamics of these changes. Single-molecule FRET-based studies indicate that AGO–miRNA complexes sample multiple conformations when incubated with target RNAs; some of these conformations are shared among different types of targets (seed pairing, seed plus supplemental pairing, and perfect pairing) whereas others are unique to one type of target (61). How these FRET-inferred conformations relate to the conformations determined by crystal structures remains to be seen.

Based on all these observations, a plausible model for formation of the TDMD-competent structure has emerged: (i) the AGO–miRNA complex binds to the trigger via seed and 3′ supplemental pairing, (ii) propagation of the 3′ pairing dislodges the 3′ end of the miRNA from AGO, which normally keeps AGO in a rigid, more extended conformation and (iii) the PAZ and N domains of the AGO protein rotate to form the TDMD-associated conformation. One or more features of this distinct conformation would thus enable recognition and selective modification of AGO–miRNA–trigger complexes by the ZSWIM8 ubiquitin ligase (and possibly other enzymes). Further work is needed to test this model by identifying molecular contacts between ZSWIM8 and the TDMD-associated conformation, and by determining the ordering and dynamics of the conformational changes prior to ZSWIM8 binding.

## What makes a good trigger RNA

Comparative genomics, reporter assays, and low- and high-throughput biochemical experiments have provided rich datasets to extract features that influence miRNA-directed target repression. Much less is known about what makes a good trigger RNA, but the growing catalog of validated trigger RNAs (Table 1) combined with a handful of mutagenesis studies are providing some early insights. Here, we are considering validated trigger RNAs to be naturally occurring RNAs (as opposed to synthetic RNAs) with detectable TDMD activity at physiological expression levels. Below, we summarize our current understanding of features that contribute to trigger RNA potency.

**Table 1.** Catalog of validated triggers. Shown are predicted pairing architectures of miRNA binding sites in naturally occurring triggers validated by knockout and/or binding site mutagenesis studies; blue shading indicates seed binding, green shading indicates 3′ pairing, and red text denotes the dinucleotides adjacent to the seed binding region. ^‡^These triggers regulate more than one miRNA within a family, but for simplicity, only one pairing diagram is shown. The type of seed binding site, the length in nts of 3′ Watson-Crick pairing and in parentheses 3′ pairing that includes G:U wobbles and one nt bulges, and the minimum free energy (MFE) in kcal/mol of 3′ pairing to nts 11 and higher in the miRNA, as determined by RNAstructure DuplexFold (103), are also provided, as well as the virus/species and reference in which validation occurred

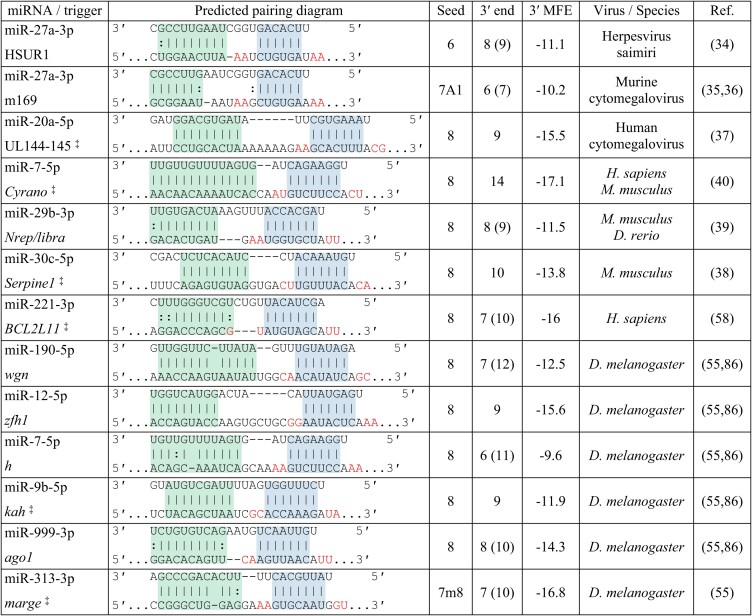

### The architecture of the TDMD-inducing site

Most TDMD-inducing sites are characterized by base-pairing to the miRNA seed, additional base-pairing to the miRNA 3′ end, and an unpaired region that joins the seed and 3′ end. Within this architecture, there is substantial diversity with respect to the length of each region, the register of the unpaired and 3′ paired regions (i.e. the first nucleotide of non-pairing or pairing, respectively, as counted from the miRNA 5′ end), and the tolerance for mismatches and wobble bases within paired regions. Consider two extremes: the *Cyrano* miR-7 site has a strong seed binding site and 14 contiguous nucleotides of 3′ pairing whereas the m169 miR-27a site has a weaker seed binding site and only six contiguous nucleotides of 3′ pairing. Both sites induce miRNA degradation although their potency (used here to describe the amount of miRNA degraded per trigger molecule) may differ (40). Nonetheless, some principles can be established based on the current set of TDMD-inducing sites.

Seed pairing is a universal feature of TDMD-inducing sites and mutations that abolish seed binding disrupt the activity of these sites (15,33–41,55,58,62). The importance of seed binding for TDMD is not surprising. Most consequential interactions between an AGO–miRNA complex and its targets are initiated via the miRNA seed (63–66). miRNA targeting is also known to follow a hierarchy of repression that strongly correlates with binding affinities between the target and the miRNA seed; 8mer sites (a perfect match to nts 2–8 of the miRNA followed by an A) are generally the most strongly repressed, followed by 7mer-M8 sites (a perfect match to nts 2–8), 7mer-A1 sites (a perfect match to nts 2–7 followed by an A), and then 6mer sites (a perfect match to nts 2–7, 3–8 or 2–6 followed by an A) and A/U dinucleotides flanking the seed site enhance both affinity for the AGO–miRNA complex and repression (67–69). Although most validated triggers have a high-affinity seed binding region, typically an 8mer site with at least one pair of flanking A/U dinucleotides (Table 1), little is known about the effect of seed binding affinity on TDMD potency. If seed binding affinity contributes to TDMD, then mutations that reduce this affinity should reduce miRNA degradation whereas mutations that enhance this affinity should increase miRNA degradation. Indeed, a *Cyrano* mutant with a 7mer-A1 site instead of the wild-type 8mer site has 75% less activity than wild-type *Cyrano* (40). This result is consistent with AGO RNA-Bind-N-Seq experiments demonstrating that miR-7 binds to 7mer-A1 sites with 10-fold lower affinity than 8mer sites (67). In contrast, HSUR1 mutants that increase seed binding affinity by extending pairing from the wild-type 6mer seed site to either a 7mer-A1 or 7mer-m8 site do not induce more miR-27a degradation than wild-type HSUR1 (15). The differential sensitivity of *Cyrano* and HSUR1 to mutations in the seed binding region indicates that features of an effective TDMD-inducing site may not be universal, perhaps influenced by miRNA sequence and/or 3′ pairing.

Another possible way to evaluate the role of seed binding affinity in TDMD is to compare the ZSWIM8 sensitivity of miRNAs that differ primarily by their seed sequences. For instance, miRNA 5′ isoforms, which are minor products generated by imprecise processing of miRNA precursor transcripts during biogenesis, have 1–2 nt 5′ extensions or truncations that often alter the seed sequence. Recently, Shi *et al.* found that 5′ isoforms of some ZSWIM-sensitive miRNAs, including miR-7, are also sensitive to ZSWIM8, which is consistent with the hypothesis that trigger RNAs may have greater tolerance for imperfect seed pairing than typical miRNA targets (57).

What sets known TDMD-inducing sites apart from other miRNA sites is extended pairing to the miRNA 3′ end. Although miRNA targets occasionally have 3–5 bp of supplemental 3′ pairing (1), 12 of 13 validated TDMD triggers are distinguished by having at least 6 bp of contiguous 3′ pairing. Extended 3′ pairing stabilizes the AGO–miRNA–target ternary complex >40-fold compared to a target harboring a seed-only site, and as discussed above, induces a distinct AGO–miRNA conformation (15). For TDMD-inducing sites, 3′ pairing frequently begins at nucleotide 12 or 13, which is also a favored register for supplemental pairing (70,71), suggesting that binding of triggers may progress from seed pairing through seed plus supplemental pairing to extended pairing.

Systematic mutagenesis of HSUR1 demonstrates that affinity to the 3′ end of the miRNA contributes to but is not the sole determinant of trigger potency (15) (Table 2). Mutations that diminish the predicted hybridization energy of 3′ pairing either reduce or abolish miR-27 degradation (15). In contrast, mutations that enhance the predicted hybridization energy of 3′ pairing (for instance, by extending pairing to the terminal nucleotides) increase the tailing of miR-27 but do not reduce the total levels of miR-27 (15). When we consider the current catalog of triggers, the predicted hybridization energy of 3′ pairing spans −9.6 to −17.1 kcal/mol (median −13.53 kcal/mol), whereas the predicted hybridization energy of 3′ supplemental pairing is typically <−5 kcal/mol (70,71). These findings are consistent with the hypothesis that a certain threshold of energy is needed to extract the 3′ end of a miRNA from its pocket in the PAZ domain and elicit a stable conformational change in the AGO–miRNA complex (15).

**Table 2.** HSUR1 site variants affect TDMD potency. Shown are predicted pairing architectures of miRNA binding sites in wild-type and mutant versions of HSUR1; blue shading indicates seed binding, green shading indicates 3′ pairing, and red text indicates the nts that have been mutated. The effects of the mutations on seed binding, 3′ end binding, TDMD, and tailing, as compared to wild-type HSUR1, are derived from Sheu-Gruttadauria *et al.*, 2019 (15) and illustrated as no change (≈, gray shading), increased (up arrow, red shading), or decreased (down arrow, blue shading)

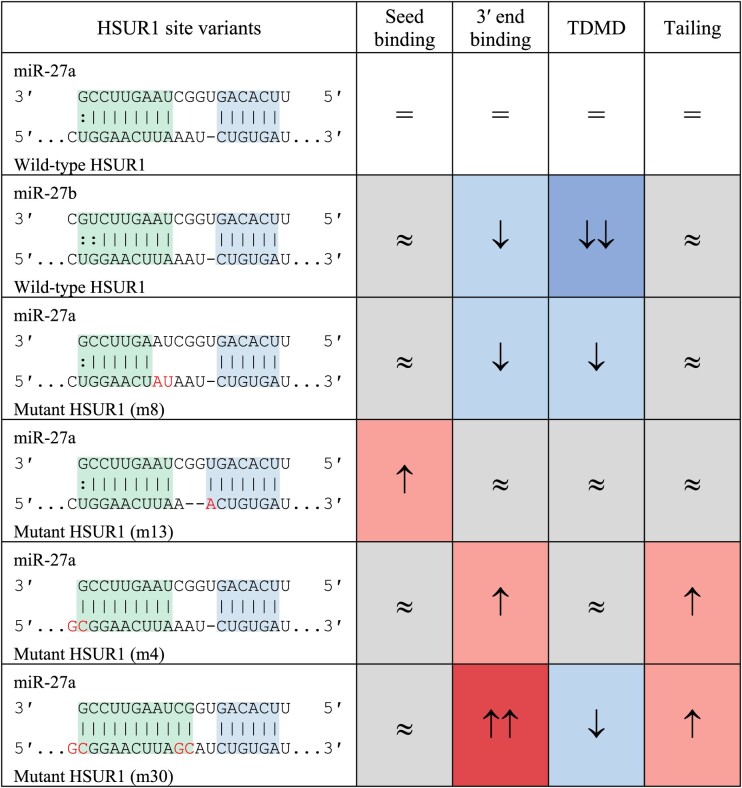

The 3′ and 5′ pairing regions are typically separated by several unpaired nucleotides (3–11 nt in validated triggers) which form a bulge or internal loop that disrupts pairing between the miRNA and trigger at positions 9 and 10, thus preventing endonucleolytic cleavage of the trigger RNA by AGO proteins capable of mediating such regulation. In addition, structural studies have shown that this bulge facilitates bending of the target RNA so that both the seed and 3′ end can pair in an A-form helix. Both loop position and loop length affect TDMD potency (15,41), likely by preventing steric clashes between the trigger RNA and the narrow central cleft of AGO, which can only accommodate an unpaired miRNA strand in the TDMD-associated conformation (15).

Although an unpaired loop is a common feature of validated TDMD sites, trigger RNAs with central complementarity can, in some instances, induce TDMD (15,33,40,42). Structures of the AGO–miRNA–trigger ternary complex demonstrate that central pairing is incompatible with a TDMD conformation, indicating that additional conformational changes are required for endonucleolytic cleavage of the target. Is the TDMD conformation of the AGO–miRNA complex in equilibrium with the endonucleolytic conformation? If so, then sliced targets may sometimes trigger miRNA degradation. Another possibility is that TDMD may be favored when a slicing target encounters a miRNA loaded into a cleavage-incompetent AGO protein. Although HSUR1-directed miR-27 degradation and Cyrano-directed miR-7 degradation act on all four human AGO proteins (15,46), we don’t yet know if TDMD induced by centrally-paired targets also acts on all AGO proteins.

Although it is tempting to assume that all TDMD-inducing sites have both seed pairing and extensive 3′ pairing, it is worth noting that these features are based on a small number of validated trigger RNAs, seven of which were initially identified by searching for features found in the other six. Indeed, it is likely that some triggers have not been found because their TDMD-inducing sites do not conform to current expectations for what a good site looks like. For example, in *C. elegans*, at least 10 miRNAs are regulated by ZSWIM8/EBAX-1, yet no triggers have been identified (46,59). Even more surprising, at least one of these ZSWIM8-sensitive miRNAs, miR-35, is still regulated even when the 3′ half of the miRNA is replaced with sequence from a ZSWIM8-insensitive miRNA (59), indicating that 3′ pairing of this miRNA with a presumptive trigger is not required for TDMD.

### Sequence context and position

While extensive pairing between the miRNA and the trigger RNA is necessary for TDMD in flies and vertebrates, there is increasing evidence that trigger RNAs can possess accessory sequences that inhibit or enhance this process. Understanding how accessory sequences tune trigger activity temporally or in specific cell types may ultimately allow us to predict these features for validated triggers and incorporate them into synthetic triggers.

For the polycistronic UL144-145 RNA from HCMV, which harbors a miR-17/20 TDMD site in its intergenic region, expression of the miRNA binding site alone induces less miRNA degradation than expression of the full-length transcript or a transcript expressing the miRNA binding site and 16 additional nts downstream of the site (37). A similar picture has emerged for some non-viral triggers. A number of validated TDMD sites, including those found in *Cyrano*, *NREP*, and *BCL2L11*, are located within larger stretches of conserved sequence. For some of these triggers, overexpression of the TDMD site causes less miRNA degradation than overexpression of the TDMD site and ∼400 flanking nucleotides (58). The authors of that study proposed that longer fragments are more likely to fold into favorable secondary structures that maintain the accessibility of the TDMD site. However, further experiments are needed to determine RNA structures of TDMD sites in their native context and the extent to which structure contributes to TDMD site potency.

Accessory sequences may also provide binding sites for proteins that regulate RNA accessibility or interact directly with AGO or the ZSWIM8 E3 ligase. Recently, Gorbea and colleagues showed that HSUR1-directed miR-27a degradation is allosterically regulated by a second miRNA, miR-142, that binds at the 5′ end of HSUR1, 33 nt proximal to the miR-27a site (72). In HeLa cells where miR-142 is not detectably expressed, HSUR1 is unable to bind or trigger the degradation of miR-27a unless miR-142 is added or the miR-142 binding site is replaced with a site for a miRNA that is normally found in HeLa cells (72). During *Herpesvirus saimiri* infection, this regulatory switch would be expected to limit HSUR1-directed miR-27a degradation to cells expressing miR-142, including most hematopoietic cells. Mechanistically, the binding of miR-142 is thought to induce a conformational change in HSUR1 that increases the accessibility of the internal miR-27a binding site. This hypothesis is consistent with *in vivo* chemical probing of HSUR1, which indicates that HSUR1 is loosely structured and dynamic in cells that express both miR-142 and miR-27a (73). The extent to which the HSUR1 structure becomes more constrained in cells that lack miR-142 and/or miR-27a still needs to be determined.

Another feature of validated trigger RNAs is the position of the TDMD site, which is typically restricted to noncoding sequences. Although some trigger RNAs such as HSUR1 and *Cyrano* are bona fide noncoding RNAs, most trigger RNAs are mRNAs that also function as TDMD triggers. For nearly all of these mRNAs, the TDMD site is positioned within the 3′ UTR, suggesting that AGO binding to TDMD sites may be displaced by scanning and translating ribosomes, consistent with what is known for typical miRNA target sites (70,74).

## Trigger RNAs shape miRNA levels and stability across diverse biological processes

### Virus–host interactions

Viruses have evolved myriad gene regulatory strategies to exploit normal host cell function and evade the immune response. One such strategy is the acute degradation of host miRNAs. Indeed, the founding example of a trigger RNA is HSUR1, an abundant 143 nt U-rich transcript produced by Herpesvirus saimiri (HVS) (34,75). HVS is an oncogenic gammaherpesvirus that infects New World primate T cells *in vivo* and transforms human T cells *in vitro* (76). During viral infection, HSUR1 binds and triggers destruction of host miR-27, a repressor of T-cell activation (15,34,77). How HSUR1-directed destruction of miR-27 and enhanced expression of activation-associated genes confer a selective advantage *in vivo* is still not clear but could enable local dissemination of HVS or promote exhaustion of antiviral host T cells.

Degradation of miR-27 is also initiated during lytic infection with murine cytomegalovirus (MCMV), a betaherpesvirus with much broader tropism than HVS (36,78). Here, the miR-27 site is embedded in the 3′ UTR of the ∼1.7 kb m169 transcript, which evolved independently from HSUR1 (35,36). Mutating the miR-27 site in MCMV attenuates viral titers in multiple mouse tissues, even in the absence of an adaptive immune system, indicating that m169-dependent degradation of miR-27 promotes viral replication in a cell-autonomous fashion (36).

miR-27 is not the only miRNA susceptible to viral TDMD. Human cytomegalovirus (HCMV), a betaherpesvirus that causes severe infections *in utero* and in immunocompromised hosts, induces degradation of miR-17 and miR-20a during lytic infection. Degradation is triggered by the ∼1.6 kb polycistronic UL144-145 transcript, which contains a miR-17/20a binding site in its untranslated intergenic region (37). Inhibiting UL144-145-dependent degradation of miR-17 and miR-20a results in delayed viral production after infection of human fibroblasts (37).

To date, all known viral trigger RNAs are produced by herpesviruses. Given the success of this strategy, it would not be surprising if other viruses have evolved RNAs that hijack TDMD to destroy host miRNAs. Thus, systematic efforts to predict and validate viral trigger RNAs are still needed.

### Cellular homeostasis and regulatory circuits

One of the first non-viral trigger RNAs to be identified, the *Cyrano* lncRNA directs miR-7 destruction in many cell types and tissues; so far, the importance of miR-7 destruction has only been explored in brain and muscle (40,79). In neurons, limiting miR-7 enables the accumulation of *Cdr1as*, a eutherian-specific circular RNA with more than 70 seed sites for miR-7 (40,80,81). Although mice lacking *Cdr1as* have impaired prepulse inhibition, and cultured *Cdr1as*-deficient hippocampal neurons display increased excitability (82,83), it remains to be seen whether the TDMD-dependent stabilization of *Cdr1as* also guards against neuronal dysfunction. In human skeletal muscle cell lines, *CYRANO*-dependent miR-7 degradation promotes myogenesis by derepressing the primate-specific miR-7 target, *MYMX*, which encodes a micropeptide important for muscle cell fusion (79).

TDMD triggers have also been linked to cell cycle control and apoptosis. In serum-stimulated mouse fibroblasts, *Serpine1*-dependent miR-30b/c degradation limits repression of miR-30 targets, including some genes implicated in cell cycle control and apoptosis (38). Loss of miR-30b/c degradation accelerates cell cycle entry into S phase and sensitizes these cells to apoptosis after serum starvation or treatment with doxorubicin. Another mRNA trigger, *BCL2L11* promotes apoptosis by two different mechanisms: 1) *BCL2L11* encodes the BH3-protein BIM, which neutralizes the anti-apoptotic BCL-2 proteins and 2) the 3′ UTR of *BCL2L11* induces degradation of miR-221/222, a miRNA family that has previously been shown to suppress apoptosis and promote cell proliferation (58). Overexpressing both the *BCL2L11* coding sequence and 3′ UTR induces apoptosis, which can be attenuated by deleting the trigger site and completely prevented by disrupting the interaction between BIM and BCL-2. In animals, *BCL2L11* induction contributes to programmed cell death of white blood cells during hematopoiesis and of neurons during development (84,85); the extent to which *BCL2L11*-dependent miR-221/222 degradation shapes these processes has not yet been explored. This curious synergy between the 3′ UTR and the coding region of the same transcript is an innovative evolutionary strategy to shift the balance towards apoptosis without relying on additional transcription. How many other trigger RNAs have coherent RNA-dependent and protein-dependent mechanisms acting on cellular homeostasis?

### Animal development, behavior and stress response

Trigger RNAs also play important roles in dynamic processes in both embryos and adults. The *marge* lncRNA (CR43432) accelerates the destruction of several miR-310 family members during Drosophila development (55). Fly embryos with mutations in the *marge* TDMD site develop cuticles with decreased integrity and fewer denticles (actin-based hook-like protrusions), which may be due to aberrant downregulation of *shavenoid*, a well-characterized miR-310 target.

Drosophila *ago1* mRNA contains a TDMD site for miR-999 in its 3′ UTR. Deletion of this site causes downregulation of miR-999 targets and reduced survival of adult flies after an oxidative stress challenge (86). The *ago1* TDMD site creates an intriguing regulatory loop in which expression of *ago1* RNA induces destruction of miR-999–AGO1 complexes while increasing overall AGO1 protein, the primary miRNA effector in Drosophila. Identifying the upstream signals that enhance AGO1 expression may offer some clues to understanding this relationship between overall miRNA activity and miR-999 levels/half-life.

The zebrafish *libra* lncRNA and its mammalian mRNA ortholog *Nrep* induce destruction of miR-29b in cerebellar granule neurons. Zebrafish lacking *libra* have increased exploratory behavior and mice with mutations in the *Nrep* TDMD site display impaired motor learning (39). *Nrep/libra* has exquisite specificity, triggering destruction of miR-29b but not its co-transcribed family members miR-29a and miR-29c, which each differ from miR-29b by 4 nt in the 3′ end. This selective restriction of miR-29b expression suggests a model in which miR-29b regulates one or more targets that are not repressed by miR-29a and/or miR-29c and that are required for granule cell function. miRNA targeting specificity driven by 3′ compensatory or supplemental pairing has been described previously (87,88).

Taken together, these findings highlight the capacity for TDMD to exert precise spatiotemporal control of miRNA expression in a variety of biological processes. As well, considering that the identification of trigger RNAs is still limited, it is likely that there are many more biological functions of trigger RNAs still to be defined.

## Methods to identify and validate miRNA–trigger RNA pairs

### Computational predictions

Although a host of bioinformatic tools are available to predict typical miRNA binding sites, these tools do not typically offer predictions for TDMD-inducing sites. For this reason, Simeone et al. developed TDMDfinder, a computational pipeline that takes miRNA sites from TargetScan (67) and scores these sites based on both quantitative and qualitative parameters—length and minimum free energy of 3′ pairing, bulge/loop length, and conservation—that are associated with validated trigger sites (62). TDMDfinder predicts 606 putative TDMD-inducing sites in humans (corresponding to 231 different miRNAs) and 521 potential TDMD-inducing sites in mice (corresponding to 209 different miRNAs) (62). The authors also showed that 20 out of 37 sites (54%) selected from these high-confidence predictions are able to induce miRNA degradation when overexpressed. Whether the 17 sites that failed to validate indicate false negatives in their overexpression assay, false positives in their predictive algorithm, or some combination of both is not clear and additional experiments are needed to demonstrate the activity of the 20 TDMD-competent sites in a physiologically relevant context. Currently, TDMDfinder predictions are limited to sites in mouse and human mRNAs.

Kingston *et al.* used similar criteria to predict TDMD sites for 9 ZSWIM8/Dora-sensitive miRNAs in Drosophila S2 cells. To validate their approach, the authors disrupted the top 1–2 predicted TDMD sites for each miRNA using CRISPR/Cas9 and measured miRNA levels; 45% (5/11) of predicted sites were experimentally validated (Table 1), while the sites that did not validate were predominantly found in lowly expressed RNAs, underscoring the importance of physiological expression levels (55). Although useful, these computational approaches do make some assumptions about what TDMD sites look like; identifying the missing TDMD sites for the remaining ZSWIM8/Dora-sensitive miRNAs may expand the criteria for what makes an effective TDMD site and enable predictions of miRNA–trigger pairs and TDMD activity in any cell type for which miRNA sequencing and RNA-sequencing is available.

### Experimental approaches

Biochemical methods that capture AGO binding sites transcriptome-wide may offer an experimental approach for identifying and distinguishing TDMD sites from other miRNA target sites. Among the most widely used are AGO-CLIP (cross-linking and immunoprecipitation) and AGO-CLASH (CLIP with ligation and sequencing of hybrids), which both involve UV crosslinking of protein to RNA in intact cells/tissue, immunoprecipitation of AGO–RNA complexes, and preparation of RNA-sequencing libraries (89–91). AGO-CLASH also introduces an intermolecular ligation step between the miRNA and target RNA that results in chimeric miRNA–target reads that provide direct evidence of identities of the RNAs within the ternary complex (90,91). These methods detect thousands of traditional miRNA binding sites as well as known TDMD sites like the HSUR-1 miR-27 site and the *Cyrano* miR-7 site (7,82,92), but can they be used alone or in combination with other features to predict new TDMD sites?

Li *et al.* used several Ago-CLASH data sets from mammalian cell lines to nominate hundreds of potential TDMD sites and then refined those predictions to 18 high-confidence TDMD sites by incorporating other features such as predicted pairing architecture, untemplated nucleotides at the 3′ end of the miRNA, and conservation (58). Three of the 18 predicted TDMD sites belonged to established triggers (*Cyrano*, *NREP* and *Serpine1*) (58). The authors also showed that 64% (7/11) of sites selected from these high-confidence predictions and 50% (1/2) of sites selected from lower-confidence predictions are able to induce miRNA degradation when overexpressed, and at least one of these predictions, the miR-221/222 site in *BCL2L11*, is also TDMD-competent in the endogenous context (58). AGO-CLASH has also been applied to ZSWIM8/Dora-deficient Drosophila S2 cells. As before, the authors identified chimeras with potential TDMD-like architecture, and then further refined the list by requiring (i) at least 4-fold more chimera reads in the Dora-deficient S2 cells than in wild-type S2 cells and (ii) at least 100 chimera reads per million in Dora-deficient S2 cells (86). These two criteria reduced the number of high-confidence TDMD sites to 5—the same 5 sites recently identified by Kingston *et al.* (55,86). Disrupting the TDMD sites using CRISPR/Cas9 validated 100% (5/5) of the high-confidence TDMD sites and 0% (0/3) of the lower-confidence TDMD sites (55,86). Given that 10 miRNAs are increased in Dora-deficient S2 cells, at least five triggers remain to be found.

### Best practices for validating trigger RNAs and miRNA substrates

Any approach that nominates miRNA-trigger RNA pairs should be complemented by experimental validation, ideally at a scale large enough to infer the extent of false positives within the overall list of predicted pairs. Here, we discuss several approaches to validate putative TDMD interactions.

**Figure 4.**
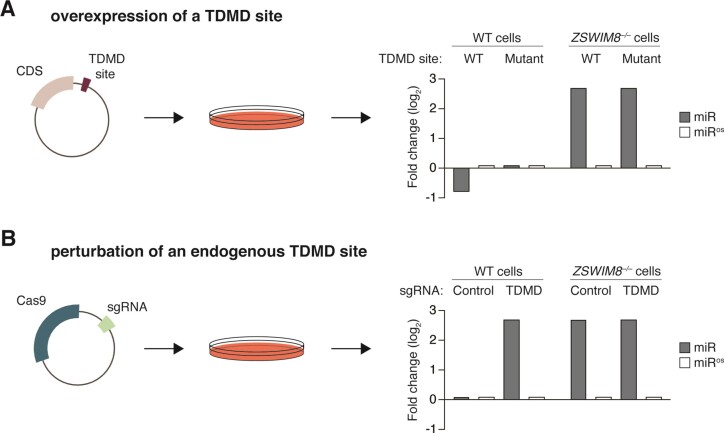
Experimental strategies to validate candidate trigger RNAs. **(A)** The most common approach for interrogating the activity of a TDMD site is depicted. The trigger RNA or a part thereof is inserted in the 3′ UTR of a reporter gene and transiently overexpressed. Here, the expectation is that the wildtype TDMD site should decrease the miRNA, whereas a mutated TDMD site should have no effect. The opposite strand of the miRNA duplex (miR^os^) should not be affected. In *ZSWIM8* KO cells, the miRNA will be increased if the endogenous trigger is normally expressed and overexpressing the wildtype TDMD site should not affect the miRNA. Fold changes are compared to WT cells expressing only the coding sequence (CDS). **(B)** The recommended approach for interrogating the activity of a TDMD site is depicted. CRISPR-Cas9 is used to disrupt the endogenous TDMD site, which should lead to an increase of the miRNA in wildtype (WT) cells. As above, the opposite strand of the miRNA duplex should not be affected. In *ZSWIM8* knockout (KO) cells, the miRNA is already increased and disrupting the TDMD site should not have any additional effect. Fold changes are compared to WT cells expressing a control sgRNA.

The most common approach for evaluating putative triggers is to overexpress the RNA or a fragment thereof and measure miRNA expression by Northern blot, RT-qPCR and/or small RNA sequencing (Figure 4A). Care must be taken to select a cell type in which the miRNA is expressed highly enough to be reliably detected. To demonstrate that a reduction in miRNA levels depends on the TDMD site, wildtype triggers should be compared to triggers with mutations within the TDMD site. Although trigger-dependent miRNA trimming and tailing may also be observed, trimming and tailing should not be used as a surrogate for TDMD since trimming and tailing are neither necessary for nor predictive of miRNA degradation.

Although potentially useful for culling nonfunctional trigger RNAs, overexpression does have a number of drawbacks. Because the trigger RNA is present at supraphysiologic levels, even a suboptimal trigger may appear effective in this assay (a false positive result). Alternatively, the common practice of using a fragment of the trigger RNA rather than the entire transcript may underestimate miRNA degradation as sequence context and *cis-*acting elements clearly enhance the TDMD activity of some triggers (a false negative result) (58,72). Furthermore, the dynamic range of this approach, at least with transient overexpression, appears to be limited to a 2–3-fold reduction in miRNA levels (40,58,62).

Demonstrating that a trigger RNA induces miRNA degradation when expressed at physiologic levels is essential for establishing that RNA as a bona fide trigger *in vivo*. This can be achieved either by knocking down/out the transcript using CRISPRi, shRNA, or CRISPR-Cas9, or preferably, by specifically mutating the endogenous TDMD site (Figure 4B). With these approaches, loss of the trigger RNA or TDMD site leads to a corresponding increase in the miRNA without impacting the other strand of the miRNA duplex or the overall miRNA pool. When knockdown/knockout approaches are used, secondary effects should be ruled out by re-expressing wild-type and TDMD-site-mutated trigger RNAs at physiologic levels.

Because ZSWIM8 is thought to be required for all TDMD, any evaluation of putative triggers should also investigate the extent to which trigger-dependent miRNA degradation depends on ZSWIM8. As seen with *Cyrano*, HSUR1 and *NREP*, bona fide trigger RNAs induce miRNA degradation in wild-type cells but not in ZSWIM8-deficient cells (46,47). Furthermore, the increase in miRNA observed with loss of the trigger RNA or TDMD site should approximate the increase in miRNA observed with loss of ZSWIM8, unless multiple trigger RNAs are acting on the same miRNA (46,47,55–58).

## Unanswered questions and concluding remarks

In this review, we’ve provided a brief history of TDMD, discussed common features of trigger RNAs, described how cells, animals, and viruses use TDMD to tune miRNA-mediated repression, and laid out technical considerations for predicting and validating miRNA–trigger pairs. Although enormous progress has been made in the past 13 years, there is still much more to learn about the mechanistic basis of TDMD, its contributions to gene regulation, and its roles in development and in disease. In this final section, we highlight some of the most pressing unanswered questions in the field.

*What is the degron recognized by ZSWIM8?* Consider the problem: <1% of all AGO–miRNA complexes in a cell are bound by trigger RNAs. How does the ZSWIM8 E3 ligase efficiently recognize and selectively ubiquitinate these AGO–miRNA–trigger complexes but not AGO–miRNA–target complexes? Structural differences between the two complexes support the hypothesis that ZSWIM8 recognizes a unique structural feature(s) of AGO–miRNA that is revealed upon trigger binding, such as the vacant 3′-nucleotide binding pocket in the PAZ domain or the rotated PAZ and N domains. Does this recognition involve ZSWIM8–AGO interactions, ZSWIM8–miRNA interactions, or both? What role, if any, do post-translational modifications of AGO, such as phosphorylation (93,94), play in this process? Whatever ZSWIM8 recognizes is likely shared among all four mammalian AGO proteins and Drosophila Ago1, which are all susceptible to TDMD, and not present in Drosophila Ago2, which is resistant to TDMD (46,95). Structural interrogation of complexes formed by diverse AGO and ZSWIM8 mutants via crystallography or CryoEM, combined with functional screening of these variants for TDMD in cells, will likely hold the key to unlocking this mystery and may also facilitate the design of TDMD-resistant AGO proteins.

*How does TDMD work in C. elegans?* Although ZSWIM8/EBAX-1 regulates the stability of at least 10 miRNAs in either embryos or adult worms (46), no trigger RNAs have been identified so far. In fact, degradation of one of these miRNAs, miR-35, requires the miRNA seed but not the miRNA 3′ end (59). Although surprising, this result is consistent with previous studies in *C. elegans* showing that 3′ pairing confers targeting specificity for miRNAs of the same seed family (87,88,96) and, in some cases, even stabilizes the miRNA (26). Because the seed sequence is required for miRNA degradation, it is likely that a target RNA is still involved—so how would a *C. elegans* trigger differ from typical target? One possibility is that *C. elegans* triggers may contain accessory sequences that directly or indirectly recruit ZSWIM8. In this scenario, the ZSWIM8 E3 ligase might even ubiquitinate AGO without needing to recognize a 3′ pairing-induced conformational change in the AGO–miRNA complex. This or other alternative mechanisms for triggering miRNA degradation need not be limited to *C. elegans* and new experimental approaches will likely be needed to identify trigger RNAs that defy the known TDMD site architecture.

*Do plants have TDMD?* Although *ZSWIM8* is only found in Bilateria, plants may have evolved a similar strategy to degrade some miRNAs. Plants have trigger-like RNAs called target mimics which were initially reported to sequester the miRNA (97). However, artificial target mimics induce miRNA degradation and this degradation depends on both the SDN exonucleases and an F-box family protein called HAWAIIAN SKIRT (HWS) (98–101). F-box proteins are substrate adapters for SCF (SKP1-CUL1-F-Box-Protein) E3 ubiquitin ligases. Further investigation is needed to determine how HWS promotes miRNA degradation and the extent to which this regulation applies to endogenous target mimics.

*Which ZSWIM8 substrate(s) matter?* Loss-of-function phenotypes in *C. elegans*, Drosophila, and mice have revealed important roles for ZSWIM8 in animal development and physiology. In each of these ZSWIM8-deficient animals, anywhere from 11 to more than 50 miRNAs are increased, and these numbers are expected to grow as more tissues and cell types are assessed. When more than one miRNA is increased, which miRNA(s), if any, drives the phenotype? We also know that ZSWIM8 has at least two other substrates, SAX-3/ROBO and DAB1, with perhaps more to be discovered. To what extent is each phenotype caused by deregulated miRNA turnover, accumulation of other ZSWIM8 substrates, or even E3 ligase-independent functions of ZSWIM8?

One way to define the contribution of individual substrates is by leveraging the power of animal genetics. In general, loss of ZSWIM8 E3 ligase activity will lead to increased abundance of substrates. Therefore, depleting (but not eliminating) individual substrates in ZSWIM8-deficient animals is expected to rescue the phenotype, whereas overexpressing individual substrates in wild-type animals will phenocopy the ZSWIM8-deficient phenotype. Although care must be taken to make sure that the levels of substrate after depletion/overexpression are physiological, these approaches have had some success. For instance, the reduced body size of ZSWIM8-deficient mice and the embryonic lethality of ZSWIM8-deficient flies are rescued, at least in part, by reducing miR-322/503 and miR-3 (55,56), respectively. These experiments indicate that TDMD and these specific miRNAs play important roles in both vertebrate and invertebrate development. In *C. elegans*, ZSWIM8 deficiency increases the frequency of axon guidance defects in worms that express a temperature-sensitive, misfolded SAX-3 protein but has no effect in worms that lack SAX-3 altogether (51).

A big challenge with these genetic approaches, especially in intact animals, is scaling up to interrogate many substrates either individually or in combination. An alternative approach is to attempt to rescue ZSWIM8-deficient phenotypes by re-expressing wild-type and mutant forms of ZSWIM8. This approach has already been used to demonstrate that interactions with Elongins B and C and HSP90 are important for the axon guidance defect discussed above (51). However, what is still needed are separation-of-function mutants that, for instance, disrupt interactions between ZSWIM8 and AGO but not interactions between ZSWIM8 and other substrates. Although challenging to create, separation-of-function mutants would greatly simplify the question of which substrate(s) matter and could even be engineered into the endogenous ZSWIM8 loci using CRISPR/Cas9.

*What are the triggers?* With >80 miRNAs regulated by ZSWIM8 (so far) and only 12 validated trigger RNAs, identification of new triggers lags behind identification of TDMD-sensitive miRNAs. Computational predictions of TDMD sites are promising but still require experimental validation, ideally by disrupting the site in its endogenous context. Comparing these TDMD site-mutant cells or animals to ZSWIM-deficient cells or animals may also help determine which miRNA substrates drive a particular phenotype. If trigger RNAs have TDMD sites with atypical architecture (longer loop lengths, noncanonical seeds, or discontiguous 3′ pairing), these are likely to be missed by current prediction algorithms. Thus, unbiased approaches to capture trigger RNAs are still needed.

**Figure 5.**
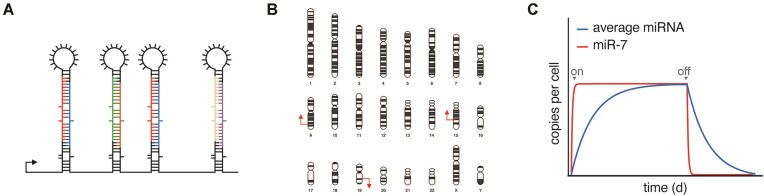
Potential advantages of regulating miRNA expression via TDMD. **(A)** TDMD can deplete select miRNAs produced from a miRNA cluster. **(B)** TDMD can globally suppress expression of miRNAs expressed by paralogous genes at distant loci (chromosome map created with BioRender.com). **(C)** Coupled with transcription (signified by gray on/off arrows), TDMD can sharpen the dynamic expression of the miRNA, thus enabling the miRNA to reach a new steady state level more quickly.

*How does TDMD fit into the spectrum of regulatory mechanisms governing miRNA expression?* Steady-state miRNA levels are a function of production and degradation rates. With so many ways to regulate miRNA production—transcription, Microprocessor-dependent cleavage of the primary transcript to generate a stem-loop, export of the stem–loop from the nucleus to the cytosol, DICER-dependent cleavage of the stem-loop to generate a miRNA duplex, and selection of the miRNA guide strand—regulating miRNA degradation might seem redundant. However, unlike regulation of transcription and Microprocessor activity, TDMD enables the decoupling of the two strands of a miRNA duplex, providing a mechanism for miRNA arm/strand switching, a phenomenon in which the dominant strand of the miRNA changes across tissues and conditions (56,57,102). This decoupling activity can also be applied to clustered miRNAs expressed from the same primary transcript (Figure 5A). Indeed, more than 60% of miRNAs regulated by ZSWIM8 are embedded within miRNA clusters (56,57). Another potential advantage of TDMD compared with other regulatory mechanisms is in managing the expression of highly similar miRNA paralogs, such as exist for miR-7, which are distributed throughout the genome (Figure 5B). Besides restricting the expression of select miRNAs, TDMD also shortens miRNA half-lives, thus enabling dynamic changes to miRNA expression (in hours instead of days) and heightening the temporal control of miRNA-mediated repression (Figure 5C). By complementing, and perhaps even coordinating with, the regulation of microRNA biogenesis, TDMD has the potential to enhance the precision and adaptability of miRNA-mediated repression. As our understanding of TDMD deepens, we will likely discover additional ways that cells and organisms harness this fascinating regulatory strategy.

## Data Availability

No new data were generated or analysed in support of this research.

## Notes

Present address: Amber F. Buhagiar, Department of Cell Biology, Albert Einstein College of Medicine, Bronx, NY 10461, USA.
